# Epigenetic Roulette in Blood Stream *Plasmodium*: Gambling on Sex

**DOI:** 10.1371/journal.ppat.1005353

**Published:** 2016-02-11

**Authors:** Andrew P. Waters

**Affiliations:** Institute of Infection, Immunity and Inflammation, College of Medical Veterinary & Life Sciences and Wellcome Trust Centre for Molecular Parasitology, University of Glasgow, Scotland, United Kingdom; Pennsylvania State University, UNITED STATES

The well-known and invidious pathology caused by malaria parasites (Plasmodium spp.) stems from the intraerythrocytic developmental cycle (IDC), which is the progressive invasion of erythrocytes by the merozoite form of the parasite followed by parasite growth, asexual replication, and lysis of the host cell liberating logarithmically greater numbers of infectious merozoites. Gene expression during the asexual IDC can largely be viewed as the result of an iterated transcriptional cascade predominantly orchestrated by members of the ApiAP2 family of transcription factors/chromatin regulators that is initiated upon sporozoite transformation in the hepatocyte [1]. However, parasite populations generated during this growth phase are far from homogeneous. Successful (even isogenic) parasite populations superimpose variation upon the basal transcriptional programme through bet hedging [2].

## What Is Bet Hedging?

Bet hedging is the process whereby certain regions of the genome (which can include multigene families, low copy number families, and single genes) are uniquely (variantly) transcribed by a small fraction of a population of cells. Each member of the population expresses its own mosaic of these variantly transcribed genes. By creating heterogeneity within the population in a host that, from the parasite’s perspective, is effectively randomly selected, the parasite population is more likely to be able to produce individuals that are able to thrive in any given host environment [2]. Seminal work from the Alfred Cortés laboratory demonstrated that bet hedging was in fact a strategy widely deployed across *Plasmodium falciparum* genomes [3]. An isolate-specific approximate 4% of each *P*. *falciparum* genome engages in variant transcription, producing significant heterogeneity and readying the population in general for varied environmental challenges. A few genes undergo variant transcription in all isolates tested, and in total, 28 gene families contain members that are variantly expressed, including all the major high-copy-number families and the well-known *var* gene family, which expresses the *P*. *falciparum* erythrocyte surface proteins PfEMP1. However, many other cell functions are putatively variant and bet hedging can include but is not limited to variation in erythrocyte binding (the *Rh* family) [4], solute uptake (*clag*3 homologues) [5], co-chaperones (*phistb*/*dnaj*) [3], and kinases (*fikk*) [3]. Regulation of variation relies heavily, if not completely, on epigenetic mechanisms and it exhibits a strong correlation with the repressive epigenetic mark of H3K9Me3, associated binding of HP1, and transcriptional variation. Further detail on the mechanism(s) that underpin bet hedging are required before it is clear whether one model would explain all forms of variant transcription, but the broad concept is valid for all variant expression.

## Epigenetic Regulation of Gene Expression and Antigenic Variation/Transcriptional Variation

Successful pathogens attempt to prolong their infection through antigenic variation—clonal expression of a single member of a structurally related but immunologically distinct repertoire of genes, e.g., *var*, which is number 60 in the typical *P*. *falciparum* genome—thereby dodging the adaptive host immune system [6]. Switching of expression between gene family members at the appropriate (low) frequency minimizes immune recognition of a parasite population that might wax and wane in numbers but persists [7]. This is a flavour of the bet-hedging strategy but one which places greater constraint on the target gene family since, for maximum success, the vast majority of the genes must be absolutely silent and have a low switching rate—constraints not necessarily applied to other variantly transcribed multi-gene families. As noted above, regulation of the *var* repertoire is achieved epigenetically, which is achieved through histone N-terminal tail modification, creating silenced zones of facultative heterochromatin [6] that are then gathered together in a filamentous actin-controlled process [8], perhaps facilitating their communal regulation. Such absolute silence might be achieved through specialised epigenetic regulation and a dedicated, and not wholly (across the genus) conserved, SET-domain containing methyltransferase (PfSet 2 or PfSet_vs_) that methylates H3K36 in *P*. *falciparum* and is associated with *var* gene silencing

*Var* genes have a particular genome organization clustering largely at the subtelomeric regions of the chromosomes, with an additional three reservoirs distributed more centromerically. Appreciating that all but one *var* will be silenced, repressed *var* are H3K9Me3-marked [9,10] and HP1-bound, which in turn promotes packaging into an extended region of silenced, condensed heterochromatin that are rendered physically more inaccessible through their spatial organization as clusters on the periphery of the nucleus. Individual *var* activation has been studied through selection of parasite clones that have switched to a specific clonotype and comparing them with those prior to the switch event. The active *var* is now marked by chromatin characterized by an acetylation motif at H3K9 (H3K9Ac), trimethylation at H3K4 (H3K4Me3), bound to nucleosomes that contain histone H2AZ and occupies a transcriptionally permissive, perinuclear location that is physically distinct from the silenced peripheral genes and characterized by the classic nucleolar protein Nop1 [11–17]. The process of activation is likely to be ordered involving first relocation, as has been seen for another variantly transcribed gene, *pfrh4*, followed by demethylation at H3K9 and, presumably, acquisition of the marks of activated chromatin [18]. Active chromatin conformation around the expressed *var* member is, at least in part, maintained by the histone methyltransferase *pfset10*, which is responsible for the H3K4Me3 mark [19].

## Transmission Is a Process Ideally Suited to Bet Hedging

**Fig 1:**
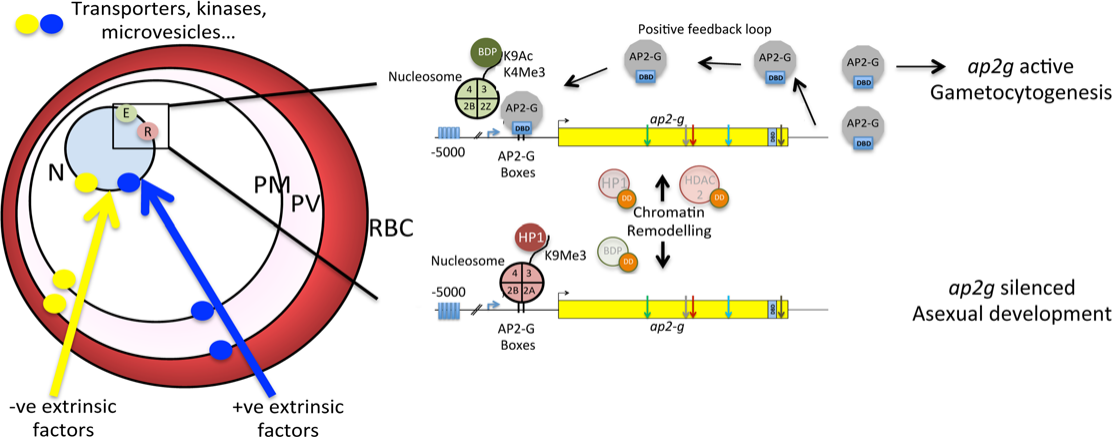
AP2-G: Scalable and bet-hedged epigenetic control of the master regulator of gametocytogenesis. Left panel: Cartoon of a malaria parasite-infected erythrocyte, showing the different compartments that are established by the parasite (PV, parasitophorous vacuole; PM, plasma membrane; N, nucleus; RBC, red blood cell), possible locations of hypothesised transporters, and kinases responding to environmental cues (e.g., microvesicles) that might subsequently influence (either positively or negatively) the gene expression/epigenetic status of variantly transcribed genes and the location of such genes at the nuclear periphery in distinct complexes and locales according to their transcriptional status (green, transcribed; red, silenced). Right panel: The yellow box represents the gene *ap2-g*, which encodes a DNA-binding transcription factor. The positions of the inactivating mutations, characterised in different lines *of P*. *berghei* that cannot produce gametocytes, are indicated. The known histone modification status and nucleosome composition associated with active and inactive genes are indicated and either inferred for this gene from evidence drawn from studies on *var* gene expression or (in)directly from epigenetic regulation and chromatin surveys. Thus, the association of HP1, which binds to H3K9me3 with inactive chromatin and silencing of *ap2-g*, is indicated, as is the association of HDAC2 activity with the generation of the active epigenetic mark, H3k9Ac. The bromodomain protein, which binds KAc in histone tails associated with active chromatin, is hypothesised here to be involved in regulating activity, but no data currently is in the public domain to support this. These three proteins have been conditionally degraded after being tagged with the shield-responsive degradation domain (DD) leading to chromatin remodelling and silencing or activation of *ap2-g*, as indicated. The proposed positive feedback loop is indicated, which explains AP2-G autoregulation, whereby AP2-G binds to its own cognate DNA-recognition motifs, which are upstream of the *ap2-g* gene in both *P*. *berghei* and *P falciparum*. DBD, DNA-binding domain; HP1, heterochromatin binding protein 1; BDP, bromodomain protein; HDAC2, histone deacetylase 2; DD, shield-regulated degradation domain.

One of the many traits that bet hedging might be applied to in the survival strategy of malaria parasites is commitment to gametocytogenesis. This is the preparation by the parasite for transmission to the mosquito, the only route by which the parasite can pass to a new host and therefore essential for long-term population survival. Gametocytogenesis is a distinct developmental pathway that generates sexually dimorphic gametocytes and bet hedging ensures that this pathway is stochastically selected by a fraction of the population. Unlike proliferating asexual blood stage forms, gametocytes are specialized, cell-cycle arrested, uninuclear forms of the parasite that circulate, and when both genders are ingested in the mosquito blood meal, they are activated to form male and female gametes that fertilise as the initial events in parasite transmission. Gametocytes have a specific half-life so the bet has to be placed each developmental cycle. Moreover, it is likely that the environmental circumstances experienced by the parasite will differ and, therefore, a more successful evolutionary strategy would be the situationally sensitive production of greater or lesser numbers of transmission forms in the optimal ratio of genders, ensuring efficient transmission to the mosquito vector (perhaps also reducing pathogenicity to a sick host). Therefore, the process of bet hedging for gametocytogenesis might be itself capable of amplification in response to environment-responsive signalling. Amplification increases the probability that any given cell in a population will commit. Such a mechanism could work because commitment to gametocytogenesis is bet-hedged on the expression of a single master regulator. A potential such regulator is the conserved transcription factor AP2-G [20,21] (Fig 1). AP2-G is a nuclear protein identified in independent studies in both *P*. *falciparum* and *P*. *berghei* (an infectious malaria parasite in rodents), and in all but one example, where the *P*. *falciparum*-specific gene *gdv1* is deleted due to a subtelomeric lesion [22], in parasite lines that have been selected for an inability to produce gametocytes, *ap2-g* contains a nonsense or missense mutation. Targeted disruption and inducible expression of *ap2-g* confirmed this role. AP2-G binds to a short eight nucleotide palindrome that is located upstream of a statistically significant number of known gametocyte-specific genes, including *ap2-g* itself (Fig 1). A model suggests itself whereby AP2-G function operates through a positive feedback loop, generating a critical threshold concentration of AP2-G that, once reached, signifies commitment and engagement with downstream promoters. One of these is AP2-G2, which is a second member of the AP2 family of transcription factors downstream of AP2-G that is essential for gametocyte maturation and contains an AP2-G binding site in its promoter region [21]. Subsequently, it has been demonstrated that AP2-G2 is responsible for a global transcriptional repression event that is an essential phase for full gametocyte maturation [23].

## *Ap2-g* Is Bet-Hedged and Epigenetically Regulated by Mechanisms with Cross Talk to Antigenic Variation

The work of Cortes’ group in describing bet hedging in *P*. *falciparum* demonstrated that the single most variantly transcribed *apiap2* family member in the genome was *ap2-g* [3]. Furthermore, two independent studies had previously identified the gene as an island of gene repression marked by H3K9Me3 [9,10] and located on the nuclear periphery [9], and a third study identified *ap2-g* as being bound by HP1 [24]. Two recent studies in *P*. *falciparum* now link these observations with AP2-G function and epigenetic regulation (Fig 1): Conditional depletion (CD) of HP1 caused a generalized decrease in the levels of H3K9Me3 mark in heterochromatin and a 50% decrease in the number of cells to enter gametocytogenesis [25]. Similarly, CD of the histone deacetylase HDAC2 increased gametocytogenesis two- to three-fold [26]. The genes up-regulated in the HDAC2CD mutants corresponded to many of those marked as being HP1-bound, coinciding with those up-regulated in HP1CD mutants in identity (if not extent) of up-regulation: Transcription of *ap2-g* was consistently up-regulated in both studies. Active epigenetic marks have not yet been described at *ap2-g*, and in wild type parasites, the majority of any population (when, in *P*. *berghei*, only a variable of approximately 10% per cycle commit to gametocytogenesis) would be expected to be in silenced heterochromatin. However, a recent report of bromodomain protein 1 (PfBDP1) binding to H3K9Ac/H3K14ac and quantitatively regulating expression of genes associated with *P*. *falciparum* merozoite invasion [27] raises the prospect of a similar stabilising action of active chromatin by an epigenetic modification binding protein that potentiates the positive feedback loop of AP2-G and kick-starts sexual development.

Given the role of bet hedging as a survival mechanism, it is perhaps not surprising that there are parallels emerging between the regulatory mechanisms for key intra-host adaptations and the single “escape mechanism” for onward transmission of the parasite.

## Perspective

We now have fascinating and tangible evidence of commonality in the mechanisms employed by the *Plasmodium* to regulate both antigenic variation and commitment to gametocytogenesis, despite the fact that they have quite different ideal goals. Through the same principle of bet-hedging (though modified by environmental sensing for *ap2-g* expression), the optimal outcomes for these two phenomena can be realised, and their detailed mechanistic comparison might provide an insight into the evolution and partitioning of these processes in *Plasmodium*. For example, the enzymatic link has not, as of yet, been ascribed to histone methylation (positive, H3K4Me3 or negative, H3K9Me3) in gametocytogenesis, and the lysine methyl transferase that is central to *var* regulation, *pfset2/pfsetvs*, is absent in the rodent malarias and therefore less likely to have a role in commitment to gametocytogenesis [28]. Ultimately, we should come to appreciate how the various aspects of the epigenetic regulatory apparatus can be layered and situationally modified by an organism in order to achieve the precise control modularity required. Bet hedging might not be achieved solely through epigenetic mechanisms. All aspects of gene expression are, in principle, amenable to stochastic variation, e.g., RNA transport, stability, translational efficiency, and protein folding—any of which might influence the level of active AP2-G, for example. The negative influence of the RNA binding protein PfPUF2 on gametocytogenesis in *P*. *falciparum* has already been described [29], and the role of Pf RNaseII in silencing of the *var* repertoire is a recent finding that might find a parallel in the control of commitment to gametocytogenesis [30]. Lastly, given that transmission to a new host is the ultimate requirement for long-term parasite survival, bet-hedged commitment to gametocytogenesis might be somehow influenced by the parasite environment. Linking the flexibility of commitment to gametocytogenesis to the sampling of the environment by the parasite seems to be an attractive strategy, although most experiments performed to address this to date do not exclude simple selection of the outcome of bet hedging. Sensing requires an apparatus to sense (cued by what, and are there multiple factors?) and a (integrating?) signalling system that could feasibly impact *ap2-g* expression (Fig 1). Concrete evidence for a sensing mechanism comes from the demonstration that exosomes or red blood cell microvesicles (RMVs) derived from infected erythrocytes influence gametocyte production in a dose-dependent manner and provide a potential link between quorum sensing and commitment to gametocytogenesis [31,32]. RMVs are biochemically complex, and the precise component(s) that might influence commitment to gametocytogenesis remains unclear. Modalities of environmental sensing, beyond quorum sensing, that allow for a direct appreciation of the suitability of an environment, existence of competing parasite genotypes, and other “threats” for which there is experimental evidence remain to be unravelled. None of the above considers the still-mysterious issue of the mechanism(s) of gender selection in gametocytogenesis, and any (or more) of the various mechanisms that have been considered [33] might prove correct. Evidence suggests that committed merozoites from the same schizont produce the same gender of gametocyte, but we lack any knowledge of the timing of gender selection, which might even precede AP2-G production.
